# The human ion channel TRPM2 modulates neuroblastoma cell survival and mitochondrial function through Pyk2, CREB, and MCU activation

**DOI:** 10.1152/ajpcell.00098.2018

**Published:** 2018-07-18

**Authors:** Iwona Hirschler-Laszkiewicz, Shu-jen Chen, Lei Bao, JuFang Wang, Xue-Qian Zhang, Santhanam Shanmughapriya, Kerry Keefer, Muniswamy Madesh, Joseph Y. Cheung, Barbara A. Miller

**Affiliations:** ^1^Department of Pediatrics, The Pennsylvania State University College of Medicine, Hershey, Pennsylvania; ^2^The Center of Translational Medicine, Lewis Katz School of Medicine of Temple University, Philadelphia, Pennsylvania; ^3^Department of Biochemistry, Lewis Katz School of Medicine of Temple University, Philadelphia, Pennsylvania; ^4^Department of Medicine, Lewis Katz School of Medicine of Temple University, Philadelphia, Pennsylvania; ^5^Department of Biochemistry and Molecular Biology, The Pennsylvania State University College of Medicine, Hershey, Pennsylvania

**Keywords:** CREB, MCU, mitochondria, neuroblastoma, Pyk2, ROS, Src, TRPM2

## Abstract

Transient receptor potential melastatin channel subfamily member 2 (TRPM2) has an essential function in cell survival and is highly expressed in many cancers. Inhibition of TRPM2 in neuroblastoma by depletion with CRISPR technology or expression of dominant negative TRPM2-S has been shown to significantly reduce cell viability. Here, the role of proline-rich tyrosine kinase 2 (Pyk2) in TRPM2 modulation of neuroblastoma viability was explored. In TRPM2-depleted cells, phosphorylation and expression of Pyk2 and cAMP-responsive element-binding protein (CREB), a downstream target, were significantly reduced after application of the chemotherapeutic agent doxorubicin. Overexpression of wild-type Pyk2 rescued cell viability. Reduction of Pyk2 expression with shRNA decreased cell viability and CREB phosphorylation and expression, demonstrating Pyk2 modulates CREB activation. TRPM2 depletion impaired phosphorylation of Src, an activator of Pyk2, and this may be a mechanism to reduce Pyk2 phosphorylation. TRPM2 inhibition was previously demonstrated to decrease mitochondrial function. Here, CREB, Pyk2, and phosphorylated Src were reduced in mitochondria of TRPM2-depleted cells, consistent with their role in modulating expression and activation of mitochondrial proteins. Phosphorylated Src and phosphorylated and total CREB were reduced in TRPM2-depleted nuclei. Expression and function of mitochondrial calcium uniporter (MCU), a target of phosphorylated Pyk2 and CREB, were significantly reduced. Wild-type TRPM2 but not Ca^2+^-impermeable mutant E960D reconstituted phosphorylation and expression of Pyk2 and CREB in TRPM2-depleted cells exposed to doxorubicin. Results demonstrate that TRPM2 expression protects the viability of neuroblastoma through Src, Pyk2, CREB, and MCU activation, which play key roles in maintaining mitochondrial function and cellular bioenergetics.

## INTRODUCTION

Transient receptor potential (TRP) channels are a superfamily of monovalent and divalent cation-permeable ion channels involved in diverse cellular functions. The TRPM (melastatin) subfamily has a number of members involved in cell proliferation and survival, including TRPM1 (15, 61), TRPM2 (8, 9), TRPM7 (1, 35), and TRPM8 (64, 77). TRPM2, the second member of this subfamily to be identified, is permeable to Ca^2+^, Na^+^, and K^+^ (54). It is widely expressed in many cell types, including brain, hematopoietic cells, and heart (16, 27, 51), and its functions under normal physiological conditions, in oxidative stress, and in ischemic injury are under investigation. Key extracellular signals that activate TRPM2 are oxidative stress (hydrogen peroxide), TNF-α, and amyloid β-peptide (18, 20, 26, 79); exposure results in production of adenosine diphosphate-ribose (ADPR), which activates the channel by binding to the TRPM2 COOH-terminal NUDT9-H domain (6, 20, 39, 51, 60, 75). In addition to ADPR, intracellular Ca^2+^ and calmodulin positively regulate TRPM2 (13, 47, 74), and acidification can inhibit channel activity (14, 72). As observed with other TRP channels, the functional TRPM2 channel is thought to be a tetramer. Four splice variants of full-length TRPM2 (TRPM2-L) have been reported, although neither their physiological roles in modulation of function nor mechanisms controlling alternative splicing are known (23, 30, 57, 79, 87). One splice variant, TRPM2-S (short, 845 residues), which is missing four COOH-terminal transmembrane domains, the Ca^2+^ pore, and the COOH terminus, can function as a dominant negative isoform and inhibit calcium influx through the full-length channel (87).

Recent publications demonstrated that TRPM2 channels are highly expressed in many cancers, including melanoma (57), breast cancer (59), prostate cancer (86), tongue cancer (89), and neuroblastoma (8), suggesting that TRPM2 may promote cell survival. In fact, TRPM2-L protected neuroblastoma cells from physiological oxidative stress, and inhibition of TRPM2 in neuroblastoma xenografts significantly reduced tumor growth and increased sensitivity to doxorubicin (5, 8). Targeting the TRPM2 channel also promoted cell death in T cell leukemia (37), gastric cancer (2), and triple-negative and estrogen-receptor positive breast cancer cell lines (38). In several other pathological models mediated through oxidative stress, TRPM2 has been shown either to promote survival, as in many cancer models (3, 28, 29, 49), or in some, enhance cell death (19, 45, 53, 69). The mechanisms and consequences of TRPM2 activation responsible for this difference need to be elucidated.

Nonreceptor proline-rich tyrosine kinase 2 (Pyk2) is an important signaling molecule that senses changes in intracellular calcium levels and translates them into alterations in cell function. Pyk2 is overexpressed in many cancers (43) and Pyk2 inhibition attenuated survival and proliferation of small cell lung cancer (66), breast cancer (80), ovarian clear cell cancer (85), multiple myeloma (88), and prostate cancer (31). Ca^2+^ influx through TRPM2 can activate Pyk2 and amplify ERK signaling in U937 cells. U937 cells are a promonocytic, human myeloid leukemia cell line isolated from a patient with histiocytic lymphoma and are often used to study differentiation of monocytes (83). Pyk2 has also been shown to modulate mitochondrial calcium uptake through phosphorylation of the mitochondrial calcium uniporter (MCU), important in mitochondrial function (57a). These data suggest that activation of Pyk2 may be a mechanism through which TRPM2 modulates cell survival. One downstream pathway by which Pyk2 activation may protect cell viability is activation of the cAMP-responsive element binding protein (CREB) (84). CREB is a key transcription factor that regulates genes involved in oncogenesis (68) and cell survival, including antioxidant genes (67). CREB has been reported to regulate mitochondrial metabolism through modulation of MCU transcription (71).

Inhibition of TRPM2 results in reduced mitochondrial function and cellular bioenergetics, increased mitochondrial reactive oxygen species (ROS), and impaired survival in ventricular myocytes isolated from both global and cardiac-specific TRPM2 knockout hearts (29, 49) and in neuroblastoma cells (5, 8). Src kinases play a key role in modulating cell viability, as well as cell proliferation, and acquisition of invasiveness (56, 66). TRPM2-mediated Ca^2+^ entry modulates Src activation, and depletion of TRPM2 prevented phosphorylation of Src at the Y416 active site (52). In turn, Src activity has been shown to be indispensable for the initial phosphorylation of Pyk2 at Y402, followed by autophosphorylation of Pyk2 required for full Pyk2 activation (66, 90). These data suggest that TRPM2 inhibition may mediate its effects on cell survival through both reduced Src activation and Pyk2 phosphorylation.

Here, to examine the link between Ca^2+^ influx through TRPM2 and preservation of tumor viability and mitochondrial function, the Ca^2+^-dependent kinase Pyk2 and downstream signaling pathways were studied. Two approaches were utilized to inhibit TRPM2 function, depletion of TRPM2 with CRISPR technology and inhibition with the dominant negative TRPM2-S isoform (8). The roles of Pyk2, CREB, and Src activation and MCU expression in modulation of neuroblastoma cell survival by TRPM2 were investigated. Our major findings are:* 1*) inhibition of TRPM2 function reduced cell viability after doxorubicin application through decreased phosphorylation and expression of Pyk2 and CREB, *2*) Pyk2 modulates CREB phosphorylation and expression downstream of TRPM2, *3*) inhibition of TRPM2 reduces phosphorylated (p)Src, phosphorylated Pyk2, Pyk2, and CREB in the mitochondria and pSrc, phosphorylated and total CREB in the nucleus, thereby impacting expression of cellular and mitochondrial genes involved in cell survival, *4*) expression and function of the mitochondrial calcium uniporter, a CREB transcriptional target activated by Pyk2 phosphorylation, were significantly reduced in TRPM2 depleted cells, and *5*) the decrease in phosphorylated Src in TRPM2-inhibited cells may contribute to reduced Pyk2 activation. Reconstitution of wild-type (wt) TRPM2 in depleted cells but not the Ca^2+^-impermeable E960D mutant restored cell viability and Pyk2 and CREB phosphorylation and expression.

## MATERIALS AND METHODS

### 

#### Depletion of TRPM2 with CRISPR and generation of stably transfected neuroblastoma cell lines.

Generation of pcDNA3.1 empty vector, TRPM2-L, and TRPM2-S stably transfected cell lines, TRPM2 CRISPR knockout (KO), and scrambled control SH-SY5Y cells was described previously (5). RT-PCR of TRPM2 in neuroblastoma cell lines to confirm TRPM2 depletion was performed as described (5).

#### Cell proliferation assay.

Cells from stably or transiently transfected cell lines or CRISPR depletion were seeded on 96-well plates and cultured in media with 250 µg/ml G418 and/or 0.5 µg puromycin, respectively, for 96 h. Cell proliferation was assessed by measurement at OD_490nm/690nm_ using XTT (2,3-Bis(2-methoxy-4-nitro-5-sulfophenyl)-2H-tetrazolium-5-carboxanilide) cell proliferation assay (Trevigen, Gaithersburg, MD) following the manufacturer’s instructions (65). In some experiments, cells were treated with doxorubicin (0.3 or 0.5 μM; Fresenius, Kabi USA, LLC, Lake Zurich, IL) for specified durations during cell culture.

#### Immunoblot analysis.

Western blotting was performed as described previously (5). Blots were probed with the following antibodies: anti-TRPM2-C (no. A-300-413-A, 1:300; Bethyl Laboratories, Montgomery, TX) (87), anti-V5-horseradish peroxidase (no. R-96125, 1:2,000; Invitrogen, Carlsbad, CA), anti-pCREB (no. 9198, 1:250; Cell Signaling Technology, Boston, MA), anti-CREB (no. 9171, 1:250; Cell Signaling Technology), anti-epidermal growth factor receptor (EGFR; no. 54359, 1:1,000; Cell Signaling Technology), anti-GAPDH (no. 2118, 1:10,000; Cell Signaling Technology), anti-lamin (no. 2032, 1:1,000; Cell Signaling Technology), anti-MCU (no. 14997, 1:500 to 1:2,000; Cell Signaling Technology), anti-pPyk2 (no. 44-618G, 1:500; Invitrogen), anti-Pyk2 (ab-32571, 1:250; Abcam), anti-SP1 (no. 5931, Specificity Protein 1; 1:1,000; Cell Signaling Technology), anti-pSrc (no. 6493, 1:2,000; Cell Signaling Technology), anti-Src (no. 2108, 1:2,000; Cell Signaling Technology), anti-Tom20 (sc-11415, 1:5,000; Santa Cruz Biotechnology), anti-tubulin (T-9026, 1:10,000; Sigma), and anti-C1ORF43 (ab-104168, 1:400; Abcam). Blots were washed and incubated with appropriate horseradish peroxidase-conjugated antibodies (NA-934 anti-rabbit, no. NA-931 anti-mouse, 1:2,000; Amersham GE Healthcare, Pittsburgh, PA). Enhanced chemiluminescence was used for detection of signal. Intensity of bands was quantitated with densitometry. In experiments in which phosphorylation was examined, blots were first probed with phospho-antibodies. Blots were then stripped and reprobed with secondary antibody and enhanced chemiluminescence. After it was determined that results were negative, and blots were reprobed with antibody to measure the total protein.

#### Inhibition of proteasome degradation.

Cells were plated and 24 h later were either untreated or treated with 0.05% DMSO or 0.5, 1, or 5 μM MG132 (Sigma). At the same time, some of the cells were treated with 0.3 μM doxorubicin for 24 h. Cells were then harvested and protein expression analyzed by Western blotting.

#### Generation of Ca^2+^-impermeable E960D TRPM2 mutant.

E960D was created using wild-type TRPM2 in pcDNA3.1V5/His vector as a template, Quick Change kit (Stratagene), and the following primers: forward 5′-CTCATCCACAACGACCGCCGGGTGGAC-3′, reverse 5′-TCCACCCGGCGGTCGTTGTGGA TGAG-3′. PCR reaction (1–2 μl of 50 μl) was used for transformation of competent DH5α (Invitrogen). Clones were verified by digestion of DNA with restriction enzymes and by sequencing.

#### Generation of wild-type and Pyk2 mutants.

Flag-tagged wild-type Pyk2 in pCMV6 entry vector was purchased from OriGene Technologies (Rockville, MD) and used as a template to amplify ∆Pyk2 using Pfu Ultra Fusion HS DNA polymerase (Agilent Technologies, La Jolla, CA) and primers, which created MluI and SgfI restriction enzymes sites as follows: forward primer 5′- CAGGAAGCGATCGCCATGCCCCAGATCCCCATGCTA-3′ and reverse primer 5′-CCGCGTACGCGTCTCTGCAGGTGGGTGGGC-3′. The construct was then purified and eluted from agarose gel using QIAquick Gel Extraction kit (Qiagen, Hilden, Germany), digested with MluI and SgfI restriction enzymes, and ligated with pCMV6 entry vector, which was also cut with MluI and SgfI restriction enzymes. MluI and SgfI restriction enzymes were from Promega (Madison, WI). Ligation mixture was diluted in TE buffer (10 mm Tris pH 7.5, mM EDTA) to 10 ng of DNA/µl, and 1 µl was used for transformation of competent DH5α (Invitrogen). Both Pyk2 and ∆Pyk2 were then transferred from pCMV6 entry vector into pCMV6-puro vector using SgF1 and Pme1 sites. Clones were verified by digestion of DNA with restriction enzymes and by sequencing. Y402F Pyk2 was created using wild-type (wt) Pyk2 in pCMV6-puro vector as a template, Quick Change kit (Stratagene) and the following primers: forward 5′-AGCATAGAGTCAGACATCTTCGCAGAGATTCCCG-3′, and reverse primer 5′- CGGGAATCTCTGCGAAGATGTCTGACTCTATGCT. PCR reaction (1–2 µl of 50 µl) was used for transformation of competent DH5α (Invitrogen). Clones were verified by digestion of DNA with restriction enzymes and by sequencing.

#### Transfection of wt Pyk2, Pyk2 mutants, and Pyk2 shRNA into neuroblastoma cell line SH-SY5Y.

Wild-type Pyk2 and Pyk2 mutant constructs were generated as described. Pyk2 shRNAs were purchased from OriGene Technologies, pCMV6-puro vector, or other constructs were transfected into either wt SH-SY5Y cells or SH-SY5Y cells stably transfected with either vector (V), L, or S isoforms of TRPM2, using the Neon Transfection System (Invitrogen) and following the manufacturer’s protocol. Cells were either used as transient transfectants or stably transfected cells as indicated. Neon transfected cells with Pyk2 shRNA, wt Pyk2, or mutant Pyk2 were cultured with 0.5 μg/μl puromycin for selection of stably transfected clones.

#### Reconstitution of TRPM2 function in TRPM2 depleted cells.

In TRPM2 reconstitution experiments, SH-SY5Y cells in which TRPM2 was depleted with CRISPR were transfected with wild-type TRPM2 subcloned into pcDNA3.1/V5 plasmid, the TRPM2 pore mutant E960D subcloned into the same plasmid (29, 82), or empty vector using the Neon Transfection System following the manufacturer’s instructions. Scrambled SH-SY5Y control cells were transfected with empty vector. Single cell clones of stably transfected cells were selected with 0.5 µg/ml puromycin and/or 600 μg/ml G418 (Geneticin, an analogue of neomycin; Gemini Bio-Products, West Sacramento, CA) and maintained in culture with 0.5 µg/ml puromycin and/or 200 μg/ml G418 as appropriate.

#### Subcellular fractionation.

Cytosol and mitochondrial fractionation was performed based on the procedure of Dr. Shey-Shing Sheu, Thomas Jefferson University, Philadelphia, PA, as follows. All procedures were done at 4°C. Approximately 80% confluent cells on 3 × 100-mm dishes were washed with isolation buffer (IB; 320, mM sucrose, 1 mM EDTA, 10 mM Tris pH 7.4), scraped in 1 ml IB per dish, and centrifuged at 700 *g* for 5 min. Cell pellets were suspended in 1.5 ml of IB with protease and phosphatase inhibitor cocktail and homogenized with Dounce homogenizer (20 strokes). Homogenate was centrifuged at 700 *g* for 10 min. Supernatant was collected and kept on ice. Pellets were suspended in 0.5 ml IB, including proteinase and phosphatase inhibitors, homogenized as before, and centrifuged at 700 *g* for 5 min. Supernatants were combined and centrifuged at 17,000 *g* for 15 min. Supernatants were kept as cytosol, and mitochondrial pellets were suspended in 100 µl of lysis buffer and incubated overnight at 4°C with rotation. Cytosol-nucleus fractionation was performed using Thermo Fisher Subcellular Protein Fractionation Kit (Rockford, IL) for cultured cells, according to the manufacturer’s protocol.

#### RT-PCR of Pyk2, CREB, and MCU.

RNA was prepared from neuroblastoma cells using RNeasy kit (Qiagen). First-strand cDNA synthesis was performed from 500 to 2,000 ng of RNA using Super Script kit (Invitrogen by Life Technologies). The cDNA was then subjected to quantitative real-time PCR reaction using 5 µl of 50× diluted first-strand cDNA reaction, Quantabio (Beverly, MA) PerfectCT SybR Green Fastmix ROX and the following primers: Pyk2 forward primer 1: 5′-CCCTCCGCAAACCAACCT-3′, Pyk2 reverse primer 1: 5′-ACCCTCAGGAACCTGGAACT-3′; Pyk2 forward primer 2: 5′-GAGAACATGGCTGACCTCATAG-3′, Pyk2 reverse primer 2: 5′-GTTCCGCTTCTCACCATCTT-3′; Pyk2 forward primer 3: 5′-AAGCCGAGTGGAGGTATGA-3′, Pyk2 reverse primer 3: 5′-GTTCCGGAGCTGTTGGTAAA-3′; CREB forward primer 1: 5′-TCACAGG AGTCAGTGGATAGT-3′, CREB reverse primer 1: 5′-CCTGGTGCATCAGAAGATAAGT-3′; CREB forward primer 2: 5′-GAACCAGCAGAGTGGAGATG-3′, CREB reverse primer 2: 5′-GGCATAGATA CCTGGGCTAATG-3′; CREB forward primer 3: 5′-CCTCTGGAGACGTACAAACATAC-3′, CREB reverse primer 3: 5′-CTCTCTTTCGTGCTGCTTCT-3′; MCU forward primer 1: 5′-GCAGAATTTGG GAGCTGTTT-3′ (63), MCU reverse primer 1: 5′-GTCAATTCCCCGATCCTCTT-3′; MCU forward primer 2: 5′-CAGTTCACACTCAAGCCTATCT-3′, MCU reverse primer 2: 5′-ATCAAGGAGGAGGA GGTCT ATT-3′; MCU forward primer 3: 5′-CTGTTGTGCCCTCTGATGAT-3′, MCU reverse primer 3: 5′-GTCAGAGATAGGCTTGAGTGTG-3′. Three sets of primers in three separate PCR reactions were used for each gene to assure the accuracy of results. Ribosomal protein l32 (Rpl32) was used as a reference gene and the primers used are as follows: Rpl32 forward primer: 5′-CATCTCCTTCTCGGCATCA-3′ and Rpl32 reverse primer: 5′-CTGGGTTTCCGCCAGTTAC-3′ (63). Real-time PCR was performed using Quantstudio 12KFlex (384 wells) or StepOne plus (96 wells) Real-Time PCR system (Applied Biosystems). Reactions were run in three or four replicates. The PCR results were analyzed using Expression Suite software (Life Technologies) as relative mRNA level of cycle threshold value using scrambled CRISPR/cas9 neuroblastoma cell line as a calibrator.

#### Measurement of MCU current and current-time integral.

Mitoplast-patch clamp recordings were performed at 30°C as previously described (36, 49).

#### Statistics.

All results are expressed as means ± SE. For analysis of protein expression levels as a function of group (vector, TRPM2-L, TRPM2-S, or Scr, KO) and doxorubicin exposure time, two-way ANOVA was used. Only when statistical significance was detected across the three (V, L, S) groups was subanalysis between any two groups (e.g., TRPM2-L vs. TRPM2-S) performed with two-way ANOVA. For analysis of MCU current (*I*_MCU_) as a function of group (Scr, KO) and doxorubicin treatment, two-way ANOVA was used. A commercially available software package (JMP Pro 13.0; SAS Institute, Cary, NC) was utilized. For other analyses, one-way ANOVA (Figs. 5, 10) or Student’s *t*-test (Figs. 4, 8) was used. In all analyses, *P* < 0.05 was taken to be statistically significant.

## RESULTS

### 

#### Inhibition of TRPM2 function in neuroblastoma reduces cell viability after doxorubicin and Pyk2 and CREB activation.

**Fig. 1.**
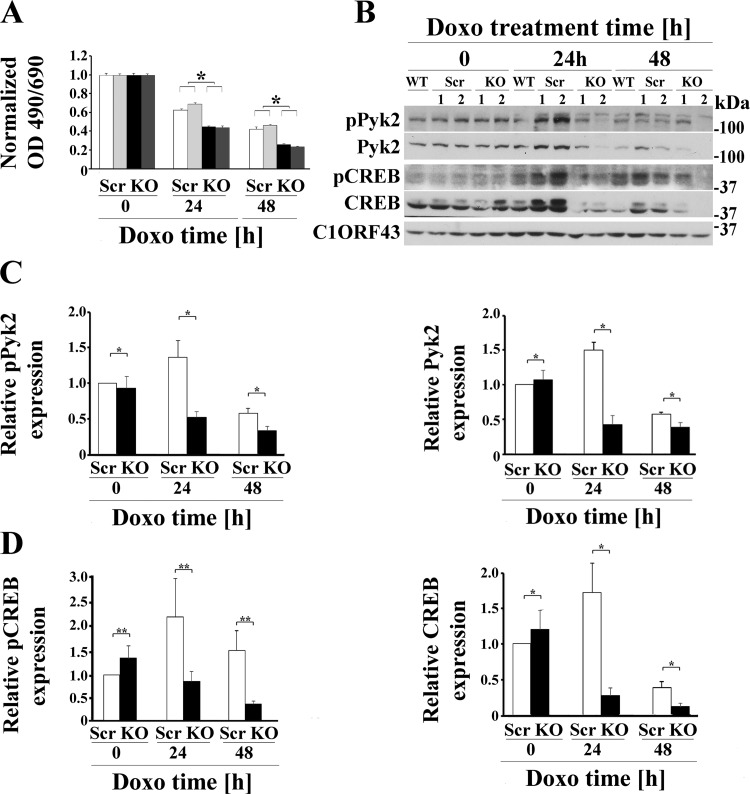
TRPM2 depletion significantly increases doxorubicin sensitivity and reduces Pyk2 and CREB phosphorylation and expression. *A*: two different SH-SY5Y clones in which TRPM2 was depleted with CRISPR (KO) or scrambled control cells (Scr) were studied. Cells were untreated or treated with 0.3 μM doxorubicin for 24 or 48 h. Cell proliferation was measured by 2,3-bis(2-methoxy-4-nitro-5-sulfophenyl)-2H-tetrazolium-5-carboxanilide (XTT) assay. Results are expressed as OD reading of plated cells (5 × 10^4^ cells) normalized to *time 0* for each group. Values are means ± SE for one representative experiment analyzed in triplicate. Four experiments were performed. *Significantly different at *P* ≤ 0.05. *B*: Western blotting was performed and phosphorylated and total Pyk2 and CREB bands were quantitated with densitometry. C10RF43 was probed to confirm equivalent loading. A Western blot of one representative experiment of four is shown. *C* and *D*: densitometry measurements from four experiments were standardized to results for each experiment’s average scrambled control at *time 0*, and the means ± SE of phosphorylated or total Pyk2 (*C*) or CREB (*D*) were calculated from four experiments are shown. *P ≤ 0.005, group × exposure time interaction effect; **P = 0.05, group × exposure time interaction effect analyzed with two-way ANOVA. CREB, cAMP-responsive element-binding protein; Doxo, doxorubicin; KO, knockout; p, phosphorylated; Pyk2, proline-rich tyrosine kinase 2; TRPM2, transient receptor potential melastatin channel subfamily member 2; WT, wild-type.

**Fig. 2.**
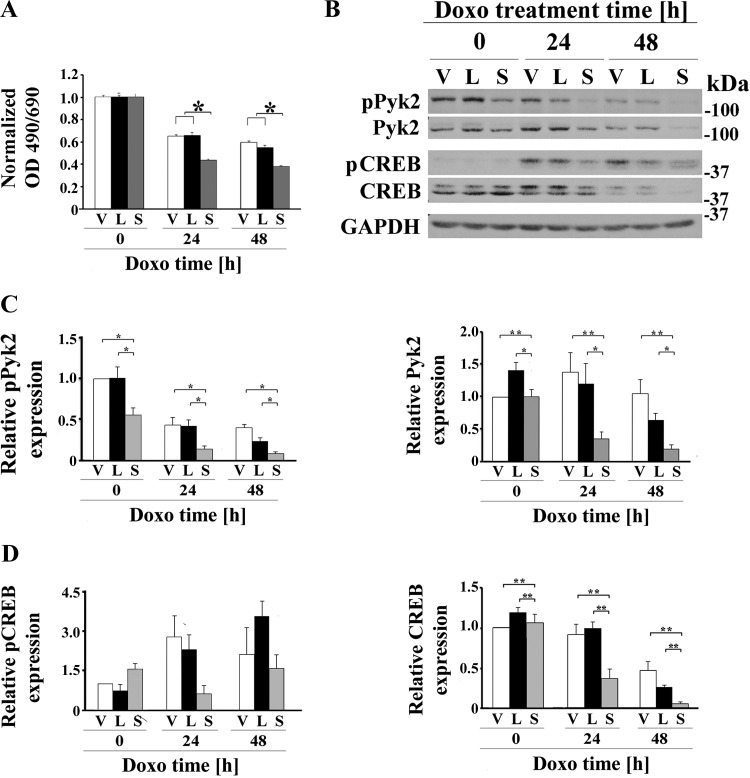
TRPM2 inhibition with TRPM2-S significantly increases doxorubicin sensitivity and reduces Pyk2 phosphorylation and Pyk2 and CREB expression. *A*: SH-SY5Y cells expressing empty vector (V), TRPM2-L (L) or TRPM2-S (S) were untreated or treated with 0.5 μM doxorubicin for 24 or 48 h. Cell proliferation was measured by 2,3-bis(2-methoxy-4-nitro-5-sulfophenyl)-2H-tetrazolium-5-carboxanilide (XTT) assay. Results are expressed as OD reading of plated cells (5 × 10^4^ cells) normalized to control at *time 0* for each group. Values are means ± SE for one representative experiment analyzed in triplicate of two performed. *Significant differences at *P* ≤ 0.05. *B*: Western blotting was performed, and phosphorylated and total Pyk2 and CREB bands were quantitated with densitometry (*B*). GAPDH was probed to confirm equivalent loading. A Western blot of one representative experiment of five (pPyk2/Pyk2) or four (pCREB/CREB) experiments is shown. *C* and *D*: densitometry measurements for each experiment were standardized to results for each experiment’s vector control at *time 0*, and the means ± SE of phosphorylated or total Pyk2 (*C*) or CREB (*D*) were calculated from five or four experiments, respectively, are shown. *P ≤ 0.0002, group effect; **P < 0.04, group × exposure time interaction effect analyzed with two-way ANOVA. CREB, cAMP-responsive element-binding protein; Doxo, doxorubicin; p, phosphorylated; Pyk2, proline-rich tyrosine kinase 2; TRPM2, transient receptor potential melastatin channel subfamily member 2; TRPM2-L, full-length TRPM2; TRPM2-S, short TRPM2.

To study the role of TRPM2 in neuroblastoma tumor growth and chemotherapy sensitivity, SH-SY5Y neuroblastoma cells in which TRPM2 was depleted with CRISPR technology (5) or cells that stably expressed the dominant negative splice variant TRPM2-S (9) were utilized. Depletion of TRPM2 and absence of ADPR-activated current were demonstrated previously (5, 8). Cells in which TRPM2 was depleted or cells expressing empty vector (V), full-length TRPM2 (L), or TRPM2-S (S) were studied without treatment or at 24 or 48 h after doxorubicin application. Doxorubicin application increases cellular ROS (22), as does TRPM2 inhibition (5). As shown previously by both XTT analysis and trypan blue exclusion, cells in which TRPM2 was depleted grew significantly slower than control cells (5). Cells in which TRPM2 was depleted (Fig. 1*A*) or inhibited by TRPM2-S (Fig. 2*A*) also had significantly increased sensitivity to doxorubicin treatment (5, 8). These studies confirm that cell viability after doxorubicin application is significantly decreased when TRPM2 function is reduced.

To explore the mechanisms responsible for increased sensitivity of TRPM2-inhibited cells to doxorubicin we examined Pyk2, which is activated by an increase in the intracellular calcium concentration through mechanisms, including calcium influx through TRPM2 (83). In neuroblastoma cells in which TRPM2 was depleted with CRISPR, both the abundance of phosphorylated Pyk2 (group × exposure time interaction effect, *P* = 0.0025) and Pyk2 expression (group × exposure time interaction effect, *P* < 0.0001) were significantly reduced with doxorubicin exposure (Fig. 1, *B* and *C*). Pyk2 has been shown to modulate neuroprotection through the Pyk2/ERK/CREB pathway (84), but the ability of TRPM2 to modulate CREB expression has not been previously demonstrated. Both phosphorylated CREB (group × exposure time interaction effect, *P* = 0.05) and total CREB levels (group × exposure time interaction effect, *P* < 0.004) were decreased after doxorubicin treatment of cells in which TRPM2 was depleted with CRISPR (Fig. 1, *B* and *D*). In five experiments, the abundance of phosphorylated Pyk2 and Pyk2 expression were also significantly reduced in cells expressing S compared with V or L (group effect, *P* < 0.0001; Fig. 2, *B* and *C*). Doxorubicin treatment resulted in significant decreases in both of phosphorylated Pyk2 (exposure time effect, *P* < 0.0001) and Pyk2 expression (exposure time effect, *P* < 0.005) across all three groups. There was significant group × exposure time interaction effect (*P* < 0.01) when comparing Pyk2 expression (Fig. 2*C*) between V and S cells, indicating doxorubicin exposure enhanced suppression of Pyk2 expression in S cells. In these neuroblastoma cells, CREB expression (group effect, *P* = 0.001; Fig. 2, *B* and *D*) but not phosphorylation (group effect, *P* = 0.43) was significantly reduced by expression of TRPM2-S. Doxorubicin exposure significantly reduced CREB expression (exposure time effect, *P* < 0.0001) but not its phosphorylation (exposure time effect, *P* = 0.067). There was significant group × exposure time interaction effect (*P* < 0.037) when comparing CREB expression between V and S cells and between L and S cells, indicating doxorubicin exposure reduced CREB expression the most in S cells.

**Fig. 3.**
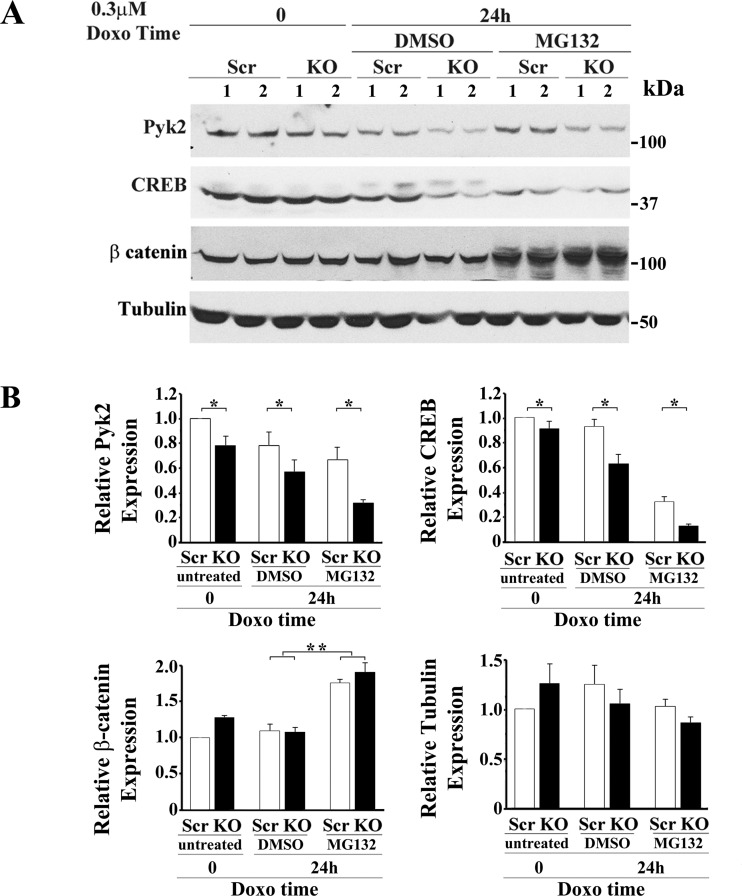
Proteasomal degradation is not the major pathway through which expression of Pyk2 and CREB are decreased in TRPM2-depleted cells after doxorubicin treatment. *A*: SH-SY5Y cells in which TRPM2 was depleted with CRISPR (KO) or scrambled control cells (Scr) were studied. Cells were treated with medium or with DMSO (vehicle) or 0.5–5 µM of the proteasome inhibitor MG132 diluted in DMSO followed by doxorubicin in four experiments. Western blots were probed with antibodies to Pyk2, CREB, β-catenin, or tubulin. Results for treatment with DMSO or 5 µM MG132, followed by 0.3 μM doxorubicin for 24 h are shown in a representative experiment. *B*: densitometry measurements for each experiment using 5 µM MG132 were standardized to results for each experiment’s scrambled control at *time 0* and analyzed with two-way ANOVA. The means ± SE of total Pyk2, CREB, β-catenin, or tubulin calculated from four (Pyk2, CREB) or three (β-catenin, tubulin) experiments are shown. *P < 0.001, group effect; **P < 0.0002, comparison of treatment with DMSO to MG132. CREB, cAMP-responsive element-binding protein; Doxo, doxorubicin; KO, knockout; Pyk2, proline-rich tyrosine kinase 2; TRPM2, transient receptor potential melastatin channel subfamily member 2.

To determine whether the decreases in total Pyk2 and CREB in TRPM2-depleted cells after doxorubicin treatment were mediated through ubiquitination and proteasome degradation, the proteasome inhibitor MG132 was added to cell culture. Cells in which TRPM2 was depleted with CRISPR or scrambled control cells were treated with medium, or with doxorubicin and DMSO or MG132 diluted in DMSO. A representative experiment is shown in Fig. 3*A*. In four experiments, Pyk2 (group effect, *P* = 0.0003) and CREB (group effect, *P* < 0.001) expression were significantly reduced in TRPM2-depleted cells compared with scrambled control cells (Fig. 3*B*). Treatment with 5 µM MG132 did not reduce the decrease in Pyk2 (*P* < 0.04) and CREB (*P* = 0.001) expression in TRPM2-depleted cells at 24 h after doxorubicin application compared with scrambled controls. This suggests that proteasome degradation does not significantly contribute to the decrease. In contrast, expression of the control protein β catenin, which is known to be regulated by proteasome degradation, was increased in both scrambled and TRPM2-depleted cells by exposure to MG132 (*P* ≤ 0.0002) and expression levels in scrambled compared with knockout cells were not different (*P* = 0.32). Expression of tubulin was not different between scrambled and knockout cells following treatment with doxorubicin or MG132 (group effect, *P* = 0.65; treatment effect *P* = 0.06).

#### Pyk2 mediates reduced expression of CREB in TRPM2-expressing cells.

**Fig. 4.**
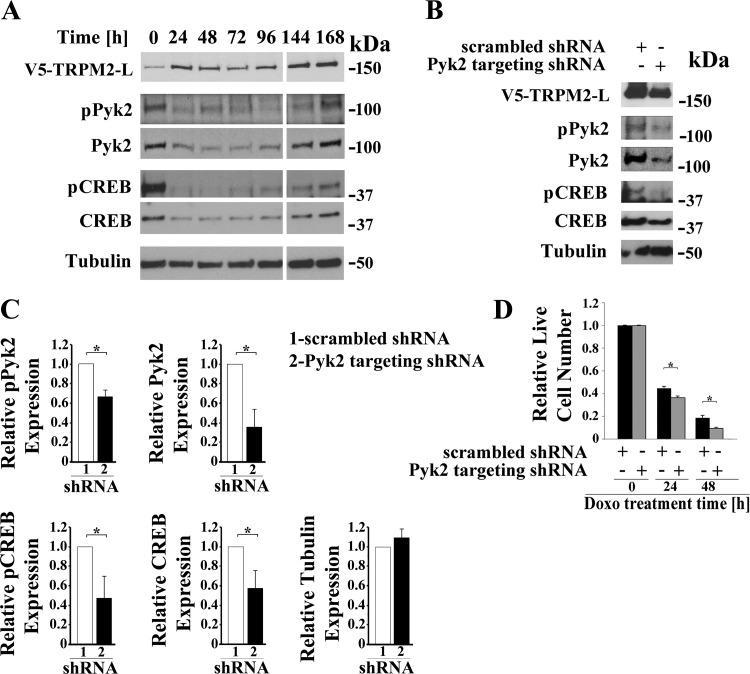
Pyk2 regulates CREB expression and cell viability. *A*: time course. SH-SY5Y cells stably expressing V5-TRPM2-L were transiently transfected with shRNA targeting Pyk2, and samples for Western blotting removed from culture at 24-h intervals for 168 h. Western blotting was performed with antibodies to V5, pPyk2, Pyk2, pCREB, and CREB. Tubulin was probed to confirm equivalent loading. *B*: SH-SY5Y cells expressing V5-TRPM2-L were stably transfected with shRNA targeting Pyk2 or control scrambled shRNA. Western blotting was performed on lysates from untreated cells with antibodies to V5, pPyk2, Pyk2, pCREB, and total CREB and confirmed that down modulation of Pyk2 resulted in reduced pPyk2, pCREB, and CREB. A representative Western blot from one of three experiments is shown. *C*: expression of pPyk2, Pyk2, pCREB, CREB, and tubulin was normalized by comparison of expression in cells transfected with Pyk2 targeted shRNA to that in scrambled shRNA for each densitometry measurement in the three experiments in *B*. The Student’s *t*-test was used for analysis of differences. *P ≤ 0.05. *D*: down modulation of Pyk2 resulted in a significant reduction in live cell number after doxorubicin in three experiments, measured with trypan blue exclusion. Measurements were standardized to results for untreated cells for each group, and the means ± SE of six replicates from one representative experiment are shown. *P ≤ 0.05. CREB, cAMP-responsive element-binding protein; Doxo, doxorubicin; p, phosphorylated; Pyk2, proline-rich tyrosine kinase 2; TRPM2, transient receptor potential melastatin channel subfamily member 2; TRPM2-L, full-length TRPM2.

To determine whether Pyk2 mediates reduced expression of CREB, Pyk2 was transiently downregulated with shRNA in SH-SY5Y cells expressing V5-tagged TRPM2-L. A time course of Pyk2 and CREB expression and phosphorylation was performed after transfection with shRNA targeting Pyk2. Pyk2 expression was reduced for 96 h (Fig. 4*A*), followed by recovery at longer times. Phosphorylated Pyk2 and CREB and CREB expression followed similar trends as Pyk2 expression.

In cells stably transfected with V5-tagged TRPM2-L and shRNA targeting Pyk2, downregulation of Pyk2 also resulted in decrease of phosphorylated Pyk2 and reduced phosphorylated and total CREB compared with cells transfected with scrambled shRNAs (Fig. 4, *B* and *C*). Expression of the control protein tubulin was not affected. Decreased Pyk2 resulted in significantly reduced live cell number after treatment with 0.3 µM doxorubicin for 24 or 48 h (Fig. 4*D*). These studies show that decreased pPyk2 and Pyk2 expression have a key role in the decreased expression and abundance of pCREB.

#### Pyk2 rescues the growth of SH-SY5Y cells expressing TRPM2-S.

**Fig. 5.**
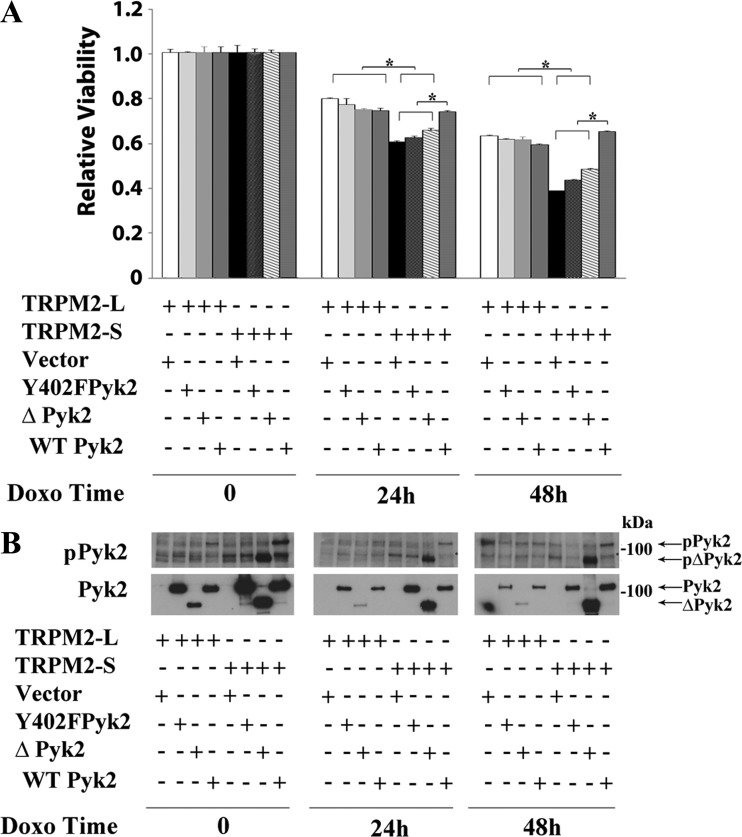
Pyk2 rescues viability of TRPM2-S expressing cells. SH-SY5Y cells expressing V5-TRPM2-L or TRPM2-S were transfected with vector, Y402F Pyk2, ∆Pyk2, or wild-type Pyk2. Cells were then treated with 0.3 μM doxorubicin for 24 or 48 h. *A*: cell proliferation was measured by 2,3-bis(2-methoxy-4-nitro-5-sulfophenyl)-2H-tetrazolium-5-carboxanilide (XTT) assay. Results are expressed as relative viability calculated by OD reading of plated cells (5 × 10^4^ cells) normalized to time 0 for each group. Values are means ± SE for one representative experiment of three done in six replicates. Results were analyzed with one-way ANOVA. *P ≤ 0.05. *B*: Western blotting was performed for all experiments to demonstrate expression of Pyk2 constructs and Pyk2 phosphorylation, and a representative blot is shown. ∆, Pyk2 mutant with deletion of N-terminal 376 amino acids; Doxo, doxorubicin; p, phosphorylated; Pyk2, proline-rich tyrosine kinase 2; TRPM2, transient receptor potential melastatin channel subfamily member 2; TRPM2-L, full-length TRPM2; TRPM2-S, short TRPM2; WT, wild-type.

To further examine the role of Pyk2 in reduced survival of cells in which TRPM2 is inhibited, cells expressing exogenous TRPM2-L or TRPM2-S were stably transfected with empty vector, Pyk2 phospho-deficient mutant Y402F (48), or wild-type Pyk2. A mutant with deletion of the NH_2_-terminal 376 amino acids, which results in high level constitutive phosphorylation at Y402, ∆376 Pyk2 was also stably transfected (48). Viability of cells was determined 24 and 48 after doxorubicin treatment with XTT. The viabilities of all TRPM2-L groups were statistically greater than all TRPM2-S groups, except for TRPM2-S transfected with wild-type Pyk2. TRPM2-S transfected with wild-type Pyk2 was statistically greater than all other TRPM2-S groups and not different from any TRPM2-L group (Fig. 5*A*). Successful transfection of these mutants and reconstitution of Pyk2 phosphorylation by wild-type Pyk2 expression in TRPM2-S expressing cells is shown in Fig. 5*B*. Endogenous Pyk2 is not seen in control cells because the exposure shown was optimized to show transfected Pyk2 expression. As reported previously (48), ∆376 Pyk2 behaved more like the mutant Y402F than wild-type Pyk2, demonstrating that autophosphorylation of Pyk2 was required but not sufficient for Pyk2-mediated preservation of cell viability by TRPM2 and suggesting a role for NH_2_-terminal Pyk2 domains.

#### Depletion of TRPM2 reduces phosphorylation of Src and Pyk2 and expression of Pyk2, CREB, and MCU in the mitochondria.

**Fig. 6.**
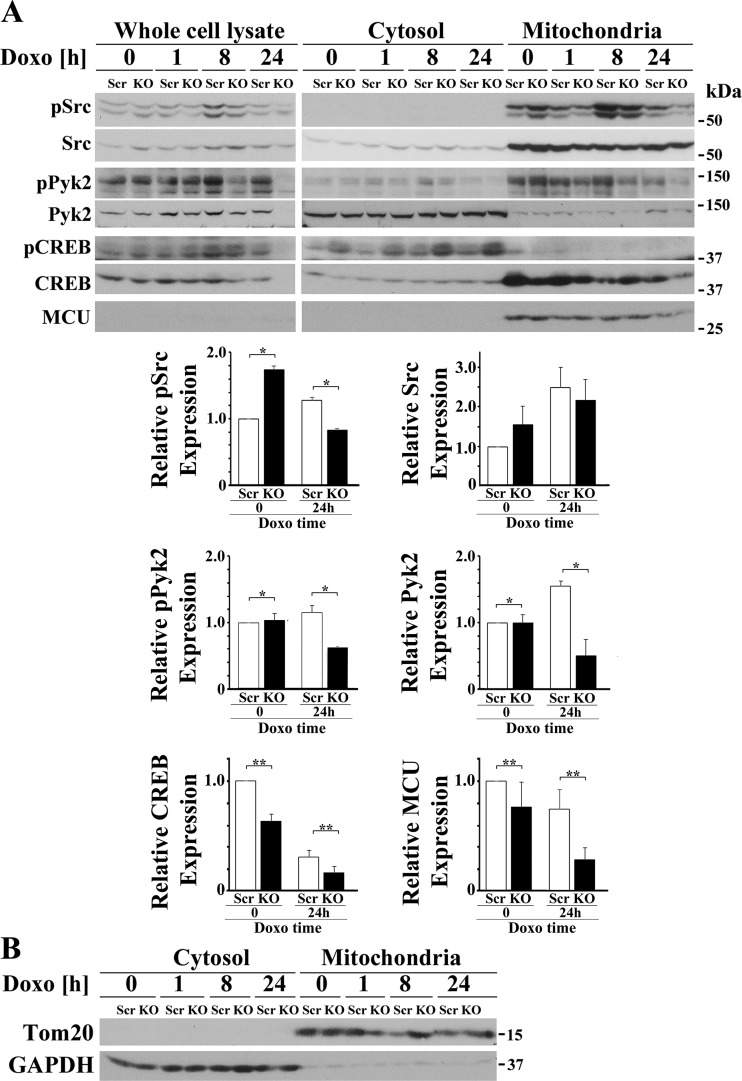
Depletion of TRPM2 reduces mitochondrial phosphorylation of Src and Pyk2 and mitochondrial expression of Pyk2, CREB, and MCU. *A*: TRPM2-depleted and scrambled SH-SY5Y cells were separated into cytosol and mitochondrial fractions and Src, Pyk2, and CREB phosphorylation and expression examined. In both whole cell lysates and mitochondria, phosphorylation of Src and Pyk2 was decreased after doxorubicin treatment of TRPM2-depleted cells. Mitochondrial Pyk2 was also decreased after doxorubicin application. Levels of CREB and the mitochondrial calcium uniporter MCU were reduced in the mitochondrial fraction of KO cells. Similar results were observed in three experiments and representative blots are shown. Densitometry measurements of mitochondrial protein for three experiments were standardized to results for each experiment’s scrambled mitochondrial control at *time 0*, and the means ± SE of phosphorylated or total Src, Pyk2, CREB, or MCU calculated from three experiments are shown. **P ≤ 0.04, group effect or *P < 0.03, group × doxorubicin exposure time interaction effect analyzed with two-way ANOVA. *B*: Western blots of Tom20 and GAPDH were done as controls for quality of separation. CREB, cAMP-responsive element-binding protein; Doxo, doxorubicin; KO, knockout; MCU, mitochondrial calcium uniporter; p, phosphorylated; Pyk2, proline-rich tyrosine kinase 2; TRPM2, transient receptor potential melastatin channel subfamily member 2.

Pyk2 is known to localize to cytosol and mitochondria (17, 57a), and recently Pyk2 was shown to phosphorylate MCU (57a). Using scrambled and TRPM2-depleted clones, SH-SY5Y cell lysates were fractionated into cytosolic and mitochondrial fractions, and phosphorylation and expression of Src, Pyk2, and CREB were examined. Representative results from one of three experiments are shown (Fig. 6*A*). Quality of cytosolic and mitochondrial separation was documented by probing blots with Tom20, which is found in mitochondria, and GAPDH, which is found in the cytoplasm (Fig. 6*B*). Although the majority of Pyk2 localized in the cytosol, phosphorylated Pyk2 was found largely in mitochondria. Src is a known activator of Pyk2, and significant Src also localized in mitochondria (90). Phosphorylated mitochondrial Src (group × doxorubicin exposure time interaction effect, *P* = 0.0228) and Pyk2 (group × doxorubicin exposure time interaction effect, *P* = 0.0035) were both decreased after 24 h of doxorubicin treatment in cells in which TRPM2 was depleted, suggesting reduced phosphorylated Src may have a role in decreased mitochondrial Pyk2 phosphorylation in TRPM2-depleted cells (Fig. 6*A*). Pyk2 but not Src was also decreased in the KO (group × doxorubicin exposure time interaction effect, *P* = 0.0066). Although a large amount of total CREB localized in mitochondria, low levels of of phosphorylated CREB were found. CREB phosphorylation is highly regulated in mitochondria and CREB phosphorylation may be transient and difficult to capture at the single time examined here (24, 25). As a group, expression of CREB (group effect, *P* = 0.0007) and MCU (group effect, *P* = 0.0393) were also significantly lower in the mitochondrial fraction of TRPM2-depleted cells. Doxorubicin treatment for 24 h significantly reduced CREB (doxorubicin effect, *P* < 0.0001) and MCU levels (doxorubicin effect, *P* = 0.0305) in both scrambled and KO mitochondrial fractions. There was insignificant group × doxorubicin interaction effect (*P* = 0.0668 for CREB; *P* = 0.4733 for MCU), indicating that doxorubicin did not affect the intrinsic differences in mitochondrial CREB or MCU expression between scrambled and KO cells. Of note, although not shown here, MCU could be seen in the whole-cell lysate when blots were probed with higher antibody concentrations and/or for longer exposure times.

**Fig. 7.**
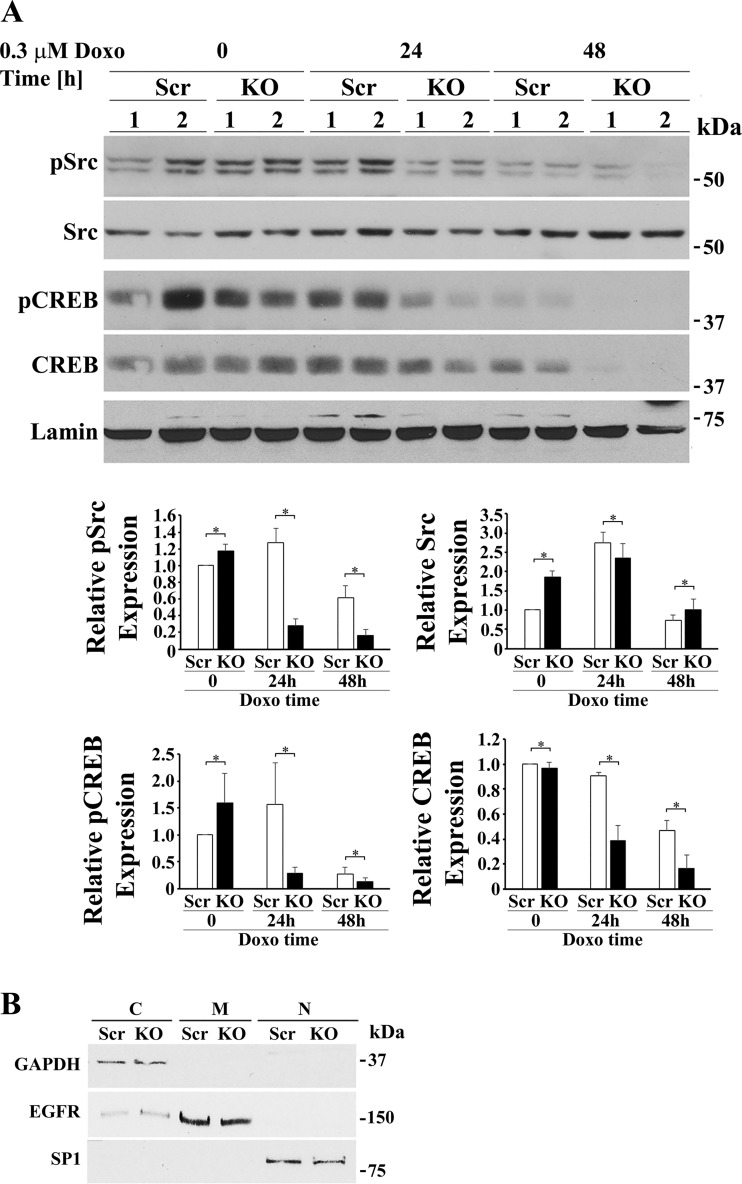
Depletion of TRPM2 followed by doxorubicin (Doxo) exposure reduces phosphorylated Src, phosphorylated CREB, and total CREB but increases total Src in the nucleus. *A*: TRPM2-depleted and scrambled SH-SY5Y control cells were treated for 24 or 48 h with Doxo and then fractionated into cytosolic and nuclear fractions. A representative Western blot from one of two experiments is shown. Densitometry measurements of nuclear protein for two experiments, each using two different clones from each group (*n* = 4), were standardized to results for each experiment’s scrambled nuclear control at *time 0*. The means ± SE of phosphorylated or total Src or CREB calculated are shown. Western blotting of nuclear fractions revealed that pSrc (group × exposure time interaction effect, *P* = 0.0002), pCREB (group × exposure time interaction effect, *P* = 0.0457), and total CREB (group × exposure time interaction effect, *P* = 0.019) were significantly reduced in the nucleus of TRPM2-depleted cells after Doxo compared with scrambled controls cells. Nuclear Src was significantly increased (group × exposure time interaction effect, *P* = 0.05) in TRPM2 depleted cells after doxorubicin treatment compared with control scrambled cells. *P ≤ 0.05, group × Doxo exposure time interaction effect analyzed with two-way ANOVA. *B*: quality of fractionation was determined by probing cytosolic (C), membrane (M), and nuclear (N) fractions with antibody to GAPDH, EGFR, and SP1, respectively. CREB, cAMP-responsive element-binding protein; EGFR, epidermal growth factor receptor; KO, knockout; MCU, mitochondrial calcium uniporter; p, phosphorylated; SP1, specificity protein 1; TRPM2, transient receptor potential melastatin channel subfamily member 2.

**Fig. 8.**
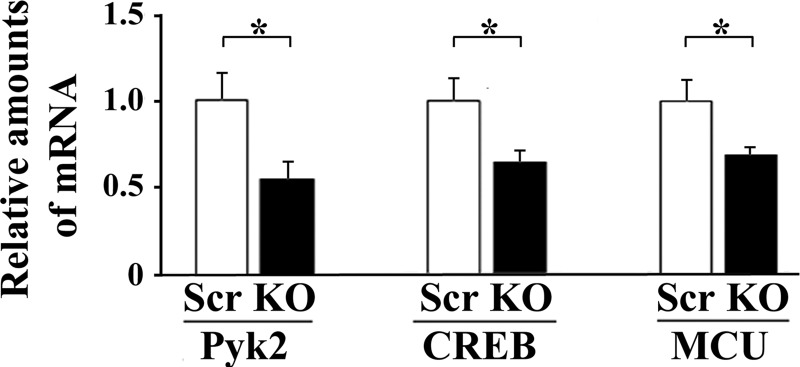
RT-PCR of Pyk2, CREB, and MCU demonstrates that these mRNAs are reduced in TRPM2-depleted cells. mRNA was prepared from scrambled control and TRPM2-depleted SH-SY5Y cells which were treated with doxorubicin for 48 h. RT-PCR was utilized to measure mRNA expression. Relative amounts were calculated compared with scrambled. *P < 0.05, statistically reduced expression of mRNA in the KO compared with scrambled analyzed in triplicate in three experiments (Student’s *t*-test). CREB, cAMP-responsive element-binding protein; KO, knockout; Pyk2, proline-rich tyrosine kinase 2; TRPM2, transient receptor potential melastatin channel subfamily member 2.

TRPM2-depleted cells were also fractionated into cytosolic and nuclear fractions. Quality of nuclear separation was documented by probing blots with lamin and specificity protein 1 (nuclear markers), epidermal growth factor receptor (membrane marker), and GAPDH (Fig. 7*B*). Western blotting of nuclear fractions revealed that both phosphorylated Src (group × exposure time interaction effect, *P* = 0.0002) and phosphorylated CREB (group × exposure time interaction effect, *P* = 0.0457) were significantly reduced in the nucleus of TRPM2-depleted cells after doxorubicin treatment compared with scrambled controls cells (Fig. 7*A*). After 24 to 48 h of doxorubicin exposure, expression of nuclear CREB (group × exposure time interaction effect, *P* = 0.019) was significantly reduced with TRPM2-depletion but not nuclear Src. Pyk2 expression in the nucleus was minimally or not detectable (data not shown). Reduction of nuclear CREB in depleted cells may play a role in the reduced expression of mitochondrial MCU and other mitochondrial proteins transcribed in the nucleus, contributing to impaired mitochondrial function. This is supported by RT-PCR analysis, which demonstrated that in TRPM2-depleted cells, mRNAs encoding Pyk2, CREB, and MCU are reduced (Fig. 8).

#### MCU current is reduced in TRPM2 depleted cells.

**Fig. 9.**
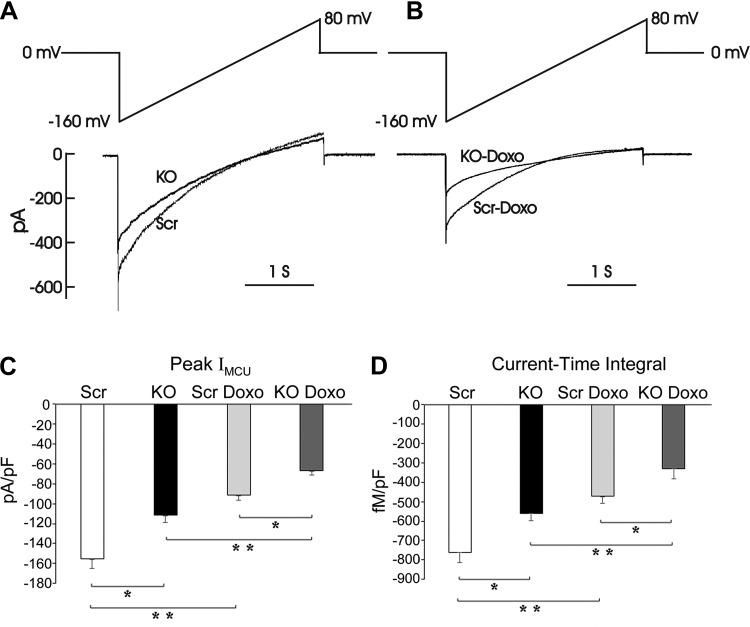
Peak mitochondrial Ca^2+^ uniporter current (*I*_MCU_) is lower in TRPM2-depleted cells. *A*: currents of mitoplasts isolated from TRPM2 KO and scrambled control SH-SY5Y cells were recorded before and after application of 5 mM Ca^2+^ to the bath medium. I_MCU_ were recorded during a voltage ramp as indicated. Traces are representative single recordings of I_MCU_ from scrambled (Scr) and knockout (KO) mitoplasts. *B*: representative *I*_MCU_ from Scr and KO mitoplasts isolated from cells after 24 h of doxorubicin exposure. *C*: peak *I*_MCU_ (pA/pF; mean ± SE) for 5 Scr and 4 KO mitoplasts, both before and after doxorubicin exposure are shown. *D*: current-time integrals indicating amount of Ca^2+^ influx during voltage ramp (fmol/pF) in Scr and KO mitoplasts from untreated cells or cells treated with doxorubicin for 24 h are shown; *P < 0.001, group effect Scr vs. KO. **P < 0.001, doxorubicin effect. Results in *C* and *D* were analyzed by two-way ANOVA. Doxo, doxorubicin; TRPM2, transient receptor potential melastatin channel subfamily member 2.

To further elucidate the mechanism of reduced mitochondrial Ca^2+^ uptake in TRPM2-depleted neuroblastoma cells (8), MCU activity in mitoplasts isolated from SH-SY5Y TRPM2-depleted cells or scrambled control cells was measured with electrophysiology. Representative currents from scrambled and KO mitoplasts recorded before and after application of 5 mM Ca^2+^ to the bath (Fig. 9*A*) or mitoplasts isolated after 24 h of doxorubicin exposure (Fig. 9*B*) are shown. In TRPM2-depleted cells, mitochondrial calcium uptake was significantly reduced because both peak mitochondrial calcium uniporter currents (pA/pF, Fig. 9*C*) and amount of calcium transported (current-time integral, Fig. 9*D*) in mitoplasts were lower (group effect, *P* = 0.0009). Doxorubicin lowered MCU current in both groups (doxorubicin effect, *P* < 0.0001) but did not affect the intrinsic differences in MCU current between KO and scrambled cells (absent group × doxorubicin interaction effect). These data suggest that decreased expression and activity of the MCU contribute to reduced mitochondrial calcium uptake in KO cells.

#### Reconstitution of TRPM2 depletion by wild-type TRPM2 but not the Ca^2+^-impermeable E960D mutant restores phosphorylation and expression of Pyk2 and CREB after doxorubicin treatment.

**Fig. 10.**
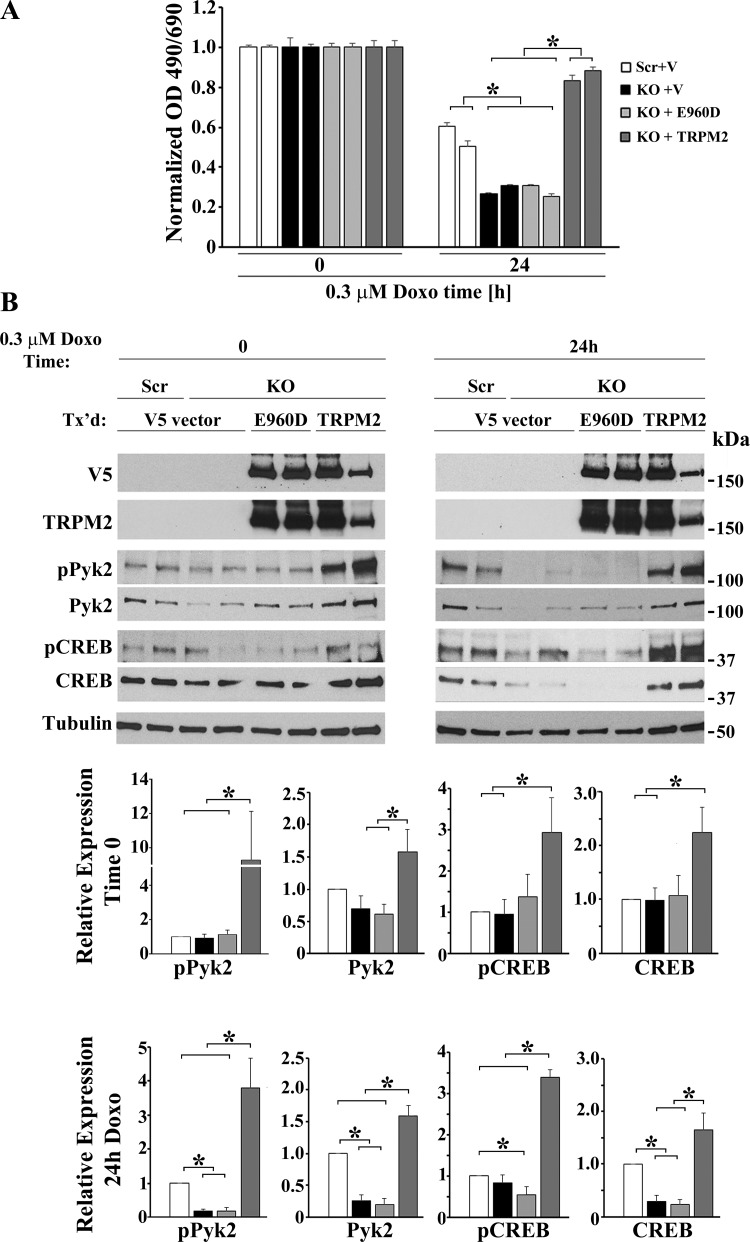
Reconstitution of TRPM2-depleted cells with wild-type TRPM2 but not the Ca^2+^-impermeable mutant E960D restored viability in TRPM2-depleted cells and phosphorylation and expression of Pyk2 and CREB. TRPM2-depleted SH-SY5Y cells (KO) were stably transfected with empty vector, V5-tagged wild-type TRPM2 or the V5-tagged Ca^2+^-impermeable TRPM2 mutant E960D. Scrambled control cells (Scr) were transfected with empty vector. Two different single cells clones from each group of transfected cells (Scr, KO) were untreated or treated with doxorubicin for 24 h and cell lysates prepared. *A*: reconstitution of cell viability with wild-type TRPM2 but not empty vector or the E960D pore mutant is shown. Viability was measured in three experiments with XTT. Measurements were standardized to results for untreated cells in each group, and the means ± SE of six replicates from one representative experiment are shown. *P ≤ 0.05. *B*: Western blotting was performed with antibodies to V5, the TRPM2 COOH terminus, phosphorylated and total Pyk2 and CREB. A representative Western blot from one of three similar experiments is shown. Results were ratioed to the average densitometry measurement of Scr control cells (*time 0* or 24 h) and statistical differences in three experiments calculated with one-way ANOVA. *P < 0.05. In TRPM2-depleted cells treated with doxorubicin for 24 h, wild-type TRPM2-L but not the E960D mutant significantly increased phosphorylation (*P* = 0.005) and expression (*P* < 0.0001) of Pyk2, as well as phosphorylation (*P* ≤ 0.02) and expression (*P* = 0.03) of CREB, when compared with vector (V) alone or E960D. In untreated TRPM2-depleted cells, reconstitution with wild-type TRPM2 but not E960D similarly significantly increased phosphorylation and expression of Pyk2 and CREB above the level found in TRPM2-depleted cells transfected with empty vector. CREB, cAMP-responsive element-binding protein; Doxo, doxorubicin; KO, knockout; p, phosphorylated; Pyk2, proline-rich tyrosine kinase 2; TRPM2, transient receptor potential melastatin channel subfamily member 2; TRPM2-L, full-length TRPM2; XTT, 2,3-bis(2-methoxy-4-nitro-5-sulfophenyl)-2H-tetrazolium-5-carboxanilide.

To determine that off target effects of knockout technology occurring during CRISPR/Cas9 treatment or subsequent selection were not responsible for these findings, SH-SY5Y cells in which TRPM2 was depleted were stably transfected with empty V5 vector, wild-type TRPM2, or the TRPM2 Ca^2+^-impermeable mutant E960D and treated with doxorubicin (29, 82). Expression of wild-type TRPM2 but not E960D preserved viability of KO cells (Fig. 10*A*), demonstrating that the reduced viability of depleted cells was due to absence of TRPM2-mediated calcium entry. In addition, expression of wild-type TRPM2 but not E960D restored phosphorylation of Pyk2 (*P* = 0.005) and CREB (*P* = 0.02) and expression of Pyk2 (<0.0001) and CREB (*P* < 0.03) in TRPM2-depleted cells treated with doxorubicin for 24 h (Fig. 10*B*). In TRPM2-depleted cells not exposed to doxorubicin, expression of pPyk2, pCREB, Pyk2, and CREB in KO cells was not statistically different from scrambled control cells expressing empty vector. In untreated TRPM2-depleted cells, wild-type TRPM2 but not E960D significantly increased phosphorylated Pyk2 (*P* < 0.02) and CREB (*P* = 0.05) and expression of Pyk2 (*P* = 0.05) and CREB (*P* < 0.02).

## DISCUSSION

TRPM2 has an important role in cell survival following oxidative stress or ischemic injury (9, 12, 49, 50). TRPM2 is also highly expressed in a number of malignancies, including melanoma, neuroblastoma, lung, and breast cancer (8, 57, 59), suggesting that it plays a role in promoting tumor growth and preserving viability. Previously, depletion of TRPM2 with CRISPR technology or inhibition with a dominant negative isoform was found to significantly impair neuroblastoma cell survival and tumor growth through modulation of mitochondrial function, mitochondrial calcium uptake, ROS production, and cellular bioenergetics (5, 8). To further explore the mechanisms, the role of the calcium-sensitive kinase Pyk2 in preserving cell viability through TRPM2 was determined. Here, inhibition of TRPM2 function was shown to decrease phosphorylation of Pyk2, CREB, and Src, and expression of Pyk2, CREB, and mitochondrial MCU. This work shows that TRPM2 maintains cell proliferation and viability through pathways involving modulation of two key kinases, Pyk2 and Src, and the transcription factor CREB, which are required to maintain MCU expression, mitochondrial calcium uptake, and mitochondrial function.

The first major finding of this work is that inhibition of TRPM2 function results in reduced viability after doxorubicin treatment through decreased phosphorylation and expression of Pyk2 and CREB. Pyk2 is a cytoplasmic tyrosine kinase activated by an increase in intracellular calcium through autophosphorylation at Y402. Pyk2 is a critical component of Ca^2+^-induced signaling pathways controlling cell survival in lung cancer (66), Src activation and metastasis in breast cancer (41), and in neuroprotection through the Pyk2/ERK/CREB pathway (84). The role of Pyk2 in the survival and proliferation of cancer cells is established (31, 43, 66, 80, 85, 88). The work here confirms the importance of calcium influx through TRPM2 in activation of Pyk2 and modulation of cell viability in cancer (83).

CREB is a nuclear-localized basic leucine zipper superfamily transcription factor that is implicated in cell survival, in the growth of many cancers, and in protection from oxidative stress by its ability to regulate a large number of antioxidant genes, reducing ROS-mediated cell toxicity (46, 62, 67, 68). Inhibition of CREB reduces proliferation and survival of tumor cells and increased CREB expression is associated with poor patient prognosis (46). The second major finding of this work is that Pyk2 is an important modulator of CREB phosphorylation and expression downstream of TRPM2 in neuroblastoma. Evidence presented here suggests that the decrease in Pyk2 and CREB expression is not secondary to ubiquitination and degradation and that reduced transcription plays a major role. CREB phosphorylation has been shown to be calcium dependent; the mechanism of reduced phosphorylation here could be through reduced Ca^2+^ entry in TRPM2-inhibited cells (81) or through reduced CREB activation downstream of Pyk2 (34, 84). Downregulation of Pyk2 with shRNA was sufficient to decrease CREB phosphorylation and expression in TRPM2-L-expressing cells (24). In addition, expression of wild-type Pyk2 restored cell viability whereas the Pyk2 phospho-deficient mutant Y402F did not, and recovery of depleted Pyk2 paralleled restoration of CREB expression and phosphorylation.

The third finding of this report is that inhibition of TRPM2 reduces mitochondrial pSrc, pPyk2/Pyk2, and CREB and nuclear pSrc, pCREB, and CREB. Through effects in the nucleus of TRPM2-depleted cells, decreased levels of pSrc and CREB, and reduced CREB-mediated transcription may contribute to decreased cell viability through lower expression of target genes involved in cell proliferation and genes involved in antioxidant defense (5, 9, 40, 62, 67).

In cell fractionation experiments performed here, we observed significant localization of CREB in the mitochondria. Phosphorylation of CREB is tightly regulated and protein phosphatase-1 rapidly dephosphorylates it at Ser-133, which could contribute to the low levels of mitochondrial pCREB seen here. CREB has an important, well-established role in the regulation of expression of mitochondrial DNA-encoded proteins, including subunits of electron transport chain complexes involved in oxidative phosphorylation (10, 11, 32). The decrease in CREB may contribute to impaired mitochondrial function, including reduced oxygen consumption rate (OCR) and ATP production observed in neuroblastoma cells in which TRPM2 was inhibited (5). Consistent with this, we previously demonstrated increased levels of mitochondrial ROS in TRPM2-inhibited cells after doxorubicin treatment and increased ROS-induced cytotoxicity (5, 8).

CREB plays an important role in mitochondrial metabolism through regulation of the expression of the mitochondrial calcium uniporter (71). Both Pyk2 and CREB are involved in regulation of MCU (57a, 71); CREB regulates its nuclear transcription and Pyk2 its mitochondrial activation. A fourth major finding of this report is that the level of MCU was significantly lower in the mitochondrial fraction of TRPM2-depleted cells after doxorubicin treatment. Reduced MCU expression in KO cells resulted in lower peak mitochondrial Ca^2+^ uniporter currents and the amount of calcium transported (current-time integral) measured in neuroblastoma mitoplasts and also observed in mitoplasts from TRPM2 knockout myocytes (49). Our data suggest that the reduced mitochondrial calcium uptake in the KO cells may be due both to decreased expression and activity of the MCU as well as reduction in the driving force for the MCU (lower mitochondrial membrane potential) reported previously (8). CREB-mediated reduction in MCU expression may be partly responsible for reduced mitochondrial calcium uptake in TRPM2-depleted cells. Low-level mitochondrial Ca^2+^ uptake is critical for normal bioenergetics and through this pathway TRPM2 inhibition may also contribute to reduced mitochondrial ATP production (7, 78). In an MCU germline (global) KO mouse, treatment of cardiac myocytes depleted of MCU with doxorubicin did not alter cardiac viability (58). However, these global MCU KOs may have developed compensatory pathways. The cardiac-specific MCU-KO had a different phenotype and showed reduced bioenergetic capacity and OCR with failure to increase mitochondrial respiration and reducing equivalents during acute stress (44). These finding are similar to findings in TRPM2 KO neuroblastoma cells. However, neither of these papers is completely reflective of our studies, in which not only is MCU significantly reduced but also a number of additional proteins due to the TRPM2 KO. The TRPM2 KO modulates expression of a wide range of proteins through reduction in transcription factors including hypoxia-inducible factor (HIF)-1/2α and CREB; not all of these have been identified. Some of these may be involved in compensatory pathways in the global MCU KO mouse. Although one pathway downstream of Pyk2 activating CREB involves ERK (4), a consistent change in ERK phosphorylation was not observed in neuroblastoma cells depleted of TRPM2 (data not shown).

Src kinases play a critical role in many aspects of cell proliferation in cancer including control of cytoskeletal organization and tyrosine phosphorylation of many signaling pathways and cytoskeletal proteins (21). Another major finding of this report is that inhibition of TRPM2 function results in decreased phosphorylation of nuclear and mitochondrial Src after doxorubicin treatment. Both Src and Pyk2 have been characterized as Ca^+^-dependent pathways (66), which may be activated by TRPM2-induced Ca^2+^ entry (52, 83). Mittal et al. (52) showed that TRPM2-dependent calcium signaling was necessary for Src activation and depletion of TRPM2 prevented Src phosphorylation at the Y416 active site, in agreement with our observations. Src was shown recently to be a priming factor, required for the initial step in Pyk2 activation, involving phosphorylation of Pyk2 at Y402 and Y579 (90). This initial phosphorylation of Pyk2 by Src is independent of Pyk2 kinase activity. Our data support the conclusion that TRPM2-mediated Ca^2+^ entry is involved in Src phosphorylation and activation, which is indispensable for Pyk2 phosphorylation and subsequently CREB activation. In addition, mitochondrial Src phosphorylates and activates mitochondrial proteins involved in mitochondrial respiration and electron transport activity, and its activity is essential for normal mitochondrial function and bioenergetics (42, 56). Thus, the decrease in phosphorylation of mitochondrial Src in TRPM2-depleted cells may also contribute to impaired mitochondrial function and high levels of ROS production (5). The decrease in nuclear Src may also influence cell proliferation and survival through pathways involving nuclear tyrosine phosphorylation, including through modulating chromatin structure (73).

**Fig. 11.**
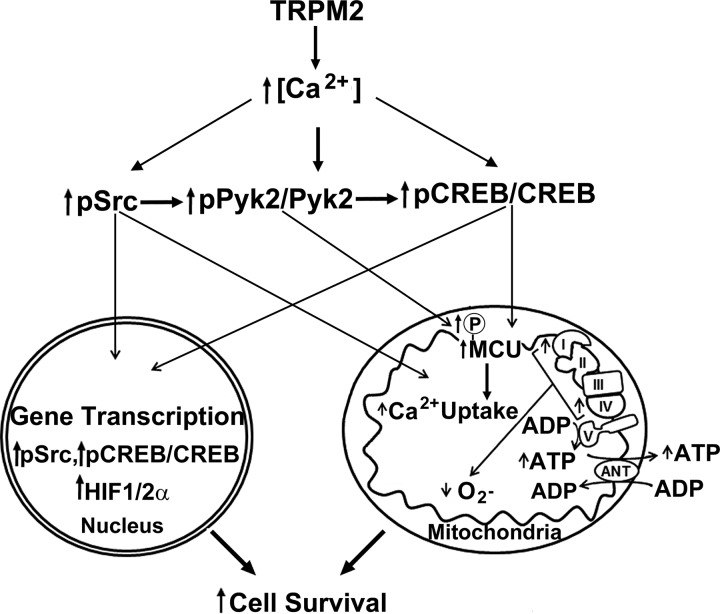
Schema of the influence of TRPM2 on mitochondrial function, ROS production, and cell survival. Intracellular calcium entry through TRPM2 results in activation of Src and phosphorylation of Pyk2, leading to increased phosphorylated and total CREB and downstream targets transcriptionally regulated by CREB, including MCU. MCU is a key modulator of mitochondrial calcium uptake. CREB contributes to maintenance of mitochondrial genes expression including those involved in electron transport. When TRPM2 is inhibited, pSrc, pPyk2, Pyk2, pCREB, CREB, and MCU are reduced and mitochondrial function and mitochondrial calcium uptake are impaired. Mitochondria with disturbed ETC and increased ROS are dysfunctional, and together with reduced anti-oxidant enzymes produce more mitochondrial and cellular ROS, enhancing susceptibility to chemotherapeutic agents and reducing cell survival and tumor growth. CREB, cAMP-responsive element-binding protein; Doxo, doxorubicin; HIF, hypoxia-inducible factor; MCU, mitochondrial calcium uniporter; p, phosphorylated; Pyk2, proline-rich tyrosine kinase 2; ROS, reactive oxygen species; TRPM2, transient receptor potential melastatin channel subfamily member 2.

Together, these results establish novel mechanisms for impaired cell survival and reduced mitochondrial function in TRPM2-inhibited cancer cells, particularly after doxorubicin treatment which increases oxidative stress (22, 76). Ca^2+^ entry through TRPM2 is shown here to be important in maintaining phosphorylation and activation of both Src and Pyk2, and CREB and MCU expression (Fig. 11). When TRPM2 function is inhibited, activation of Src is reduced and phosphorylation of Pyk2 is decreased, both by reduced calcium entry and decreased Src priming. Downstream of Pyk2, expression of CREB and its downstream target genes are impaired. Expression of MCU, a nuclear CREB transcription target, is reduced, whereas decreased Pyk2 impairs MCU activation. The reduction in MCU and mitochondrial Ca^2+^ entry, together with decreased HIF-1α, CREB, and pSrc, and the mitochondrial proteins they regulate, including members of the electron transport chain (5), combine to severely impair mitochondrial function, increase ROS, and reduce cell viability. Evidence increasingly supports the novel therapeutic approach of targeting TRPM2 to reduce tumor proliferation and survival in a number of malignancies, including neuroblastoma.

## GRANTS

This work was supported in part by National Institutes of Health Grants R01-GM-117014, R01-DK-108185, RO1-HL-123093, RO1-DK-46778, and RO1-HL-137426; Hyundai Hope on Wheels; and the Four Diamonds Fund of the Pennsylvania State University.

## DISCLOSURES

No conflicts of interest, financial or otherwise, are declared by the authors.

## AUTHOR CONTRIBUTIONS

I.H.-L., M.M., J.Y.C., and B.A.M. conceived and designed research; I.H.-L., S.-j.C., L.B., J.W., X.-Q.Z., S.S., K.K., and B.A.M. performed experiments; I.H.-L., J.Y.C., and B.A.M. analyzed data; I.H.-L., J.Y.C., and B.A.M. interpreted results of experiments; I.H.-L. and B.A.M. prepared figures; I.H.-L. and B.A.M. drafted manuscript; I.H.-L., S.-j.C., M.M., J.Y.C., and B.A.M. edited and revised manuscript; I.H.-L., S.-j.C., L.B., J.W., X.-Q.Z., S.S., K.K., M.M., J.Y.C., and B.A.M. approved final version of manuscript.
